# Breast cancer screening and its associating factors among hungarian women aged 45–65: a cross-sectional study based on the European health interview surveys from 2009 to 2019

**DOI:** 10.1186/s12889-023-16608-5

**Published:** 2023-08-31

**Authors:** Jenifer Pataki, Viktor Dombrádi, Attila Sárváry, Gergő József Szőllősi

**Affiliations:** 1 Faculty of Health Sciences, University of Debrecen, Debrecen, Hungary; 2 Doctoral School of Health Sciences, University of Debrecen, Debrecen, Hungary; 3 Health Services Management Training Centre, Faculty of Health and Public Administration, Semmelweis University, Budapest, Hungary; 4 Department of Integrative Health Sciences, Faculty of Health Sciences, University of Debrecen, Debrecen, Hungary; 5 Coordination Center for Research in Social Sciences, Faculty of Economics and Business, University of Debrecen, Debrecen, Hungary

**Keywords:** Breast cancer, EHIS, Epidemiology, Mammography, Screening

## Abstract

**Background:**

In 2020, globally 685,000 people died, and 2.3 million women were diagnosed with breast cancer. The main cause of cancer deaths among women is breast cancer, which account for 15.5% of all cancer deaths. Most of these could have been avoided with timely diagnosis.

The aim of our study was to determine the proportion of breast screening participation in Hungary, and to identify possible factors that may influence breast screening attendance.

**Methods:**

Our data were gathered from the cross-sectional European Health Interview Surveys conducted in Hungary in 2009, 2014, and 2019. In terms of categorical characteristics, Pearson’s chi-square test was performed to evaluate the differences between people who have attended breast screening within two years and who have only attended more than two years ago. To determine the factors that may have an impact on the uptake of screening, generalized linear model with logit link function regarding binomial probability distribution was executed.

**Results:**

The responses of 2626 women between the age 45-65 were included in our study. In 2009 85% (*n*=741), in 2014 90% (*n*=851) and in 2019 87% (*n*=699) of the respondents claimed to have ever attended a breast screening in their life. In 2009 68% (*n*=594), in 2014 66% (*n*=630) and in 2019 64% (*n*=515) said that they have taken part in breast screening within two years (*p*=0.331). From 2014 to 2019 (AOR=0.72 [0.57-0.89]) the chance of attending breast screening was decreasing. We observed that both secondary (AOR=1.97 [1.60-2.44]) and tertiary educational level (AOR=2.23 [1.67-3.00]), higher perceived income (AOR=1.54 [1.25-1.90]), and more frequent meeting with the doctor (AOR=1.77 [1.39-2.27]) and with the specialist (AOR=1.88 [1.54-2.28]) appeared as protective factors of breast screening attendance.

**Conclusions:**

Our results show that the lifetime prevalence of breast screening participation is high, however the recommended biennial rate is relatively low. To increase the participation rate, various initiatives would be needed, especially for women in identified risk groups, which are lower educational level, lower perceived income, and less frequent meeting with the doctor and with the specialist.

## Background

### Breast cancer statistics

In 2020, 685,000 people worldwide died due to breast cancer and 2.3 million women were diagnosed with it [1–4]. The primary causes of cancer death in women are breast and cervical cancer, which account for 15.5% and 7.7% of all cancer fatalities, respectively [1–3]. The most common cancer in the globe as of the end of 2020 was breast cancer, which had been diagnosed in 7.8 million women in the previous five years. In comparison to other types of cancer, breast cancer causes more women to lose disability-adjusted life years (DALYs) globally. In any country in the world, breast cancer can strike a woman after puberty at any age; however, it tends to strike more frequently in later life. The prognosis for breast or cervical cancer is considerably improved by early identification. Taking part in a cancer screening program is linked to an 89% decrease in cervical cancer mortality and a 21–25% decrease in breast cancer mortality [1, 5–7]. The WHO Global Breast Cancer Initiative (GBCI) aims to prevent 2.5 million breast cancer deaths worldwide between 2020 and 2040 by reducing the annual global breast cancer mortality rate by 2.5%. If breast cancer death rates were reduced by 2.5% per year, 25% of breast cancer deaths by 2030 and 40% by 2040 could be avoided in women under 70 [8, 9].

The incidence of malignant tumors is increasing in Hungary as well. The overall breast cancer mortality rate in Hungary is currently in third place, similar to that of more developed countries. In order to reduce the burden of cancer, huge efforts have been made in both diagnostics and therapy. Since 2001, Hungary has conducted organized cancer screenings [10].

### Breast cancer screening

The aim of screening is to detect tumors and precancerous conditions that are not yet causing symptoms and complaints, to prevent more serious consequences later. Breast cancer screening tests are mammogram (X-ray) and Magnetic Resonance Imaging (MRI). Mammograms are for many women the best technique to detect breast cancer at an early stage, when it is less difficult to cure and before it becomes large enough to feel or produce symptoms. Regular mammograms can reduce the risk of breast cancer-related death. For most women who are of screening age, mammography is the best method of detecting breast cancer. A breast MRI takes images of the breast using radio waves and magnets. Women who have a high risk of developing breast cancer are screened using a breast MRI in addition to mammography. For women at average risk, breast MRIs are not used since they may show abnormalities even in the absence of cancer [11, 12].

A straightforward and affordable screening method that shows promise is clinical breast examination (CBE). According to the Canadian National Breast Screening Study’s 25-year follow-up findings, adding yearly mammograms to physical exams or standard care does not further reduce breast cancer mortality in women aged 40 to 59 years in the current scenario with adjuvant therapy available for the management of breast cancers. Three RCTs on CBE screening versus no screening found that CBE had a smaller impact on tumor detection and downstaging than no screening [13].

The least expensive technique is breast self-examination. It only has a chance of lowering mortality if it is done well and is supported by the necessary diagnostic follow-up. However, field research reveals that women are frequently highly irregular and forget the strategy even after receiving thorough health education [13].

The United States Preventative Services Task Force (USPSTF) is a group of medical professionals and disease specialists who review research on the most effective methods of disease prevention and offer suggestions on how doctors can assist patients avoid illnesses or detect them early. The USPSTF advises mammograms every two years for women between the ages of 50 and 74 who are at average risk for breast cancer. Women between the ages of 40 and 49 should discuss the timing and frequency of mammograms with their doctor or other health care professional. Before starting mammograms before the age of 50, women should consider the advantages and disadvantages of screening testing [12].

According to The European Commission Initiative on Breast Cancer (ECIBC) women aged 45–49 should attend breast screening in every two or three years, from 50 to 69 years in every two years, and women aged 70–74 should participate in every three years. The organized screening programs are for women who are asymptomatic and have an average risk of breast cancer [14, 15].

Since 2002, breast screening in Hungary has been provided on an organized basis - every 2 years - for women aged 45–65 years who are eligible according to the National Health Insurance Fund register, based on Decree 51/1997 (XII.18) NM. Women aged between 45 and 65 who have not had a breast examination publicly funded by the National Health Insurance Fund within two years of the scheduled date of the screening will receive an invitation letter for public health breast screening with the assistance of the National Public Health Center from the mammography center of their place of residence [16]. Breast screening consists of a physical examination and mammography [16, 17].

### Aims

The aim of our study was to determine the proportion of breast screening attendance in Hungary, and to identify possible factors that may influence breast screening participation.

## Methods

### Database

Our data were gathered from the cross-sectional European Health Interview Surveys conducted in Hungary in 2009, 2014, and 2019 on representative samples using a standardized questionnaire under the supervision of Eurostat. The European Health Interview Survey was carried out based on stratified two-step probability samples selected to produce precise estimates of health status indicators for the Hungarian population aged 15 and older living in private households. All the three databases are representative of the Hungarian population [18].

### Data

Our study’s main outcome was focused on the uptake of biennial breast screening based on self-declaration. We used an indicator for the year of primary data collection (2009, 2014 or 2019). There were information about the respondents’ socio-demographic characteristics, such as type of residence (urban/rural), marital status (has a partner/married or has no partner), perceived income (good or bad) and highest educational level (primary, secondary, or tertiary). Children in Hungary attend primary school from the age of six to fourteen, often in classes one through eight, which was considered primary education. Then, from the ages of 14 to 18, the children attend secondary school, where they can also earn a vocational qualification, which was regarded as a secondary educational level. A university diploma or other equivalent qualification was the highest level of education, known as tertiary. The analysis also included self-perceived health status responses (good or bad). Responses to the query “How much can you do for your health?“ were also used (not so much or a lot). We analyzed the existence of any self-reported chronic health problem (have/not have) and healthcare-related variables as well, like the most recent visit to a doctor (within 12 months/more than 12 months) and to a specialist (within 12 months/more than 12 months). Furthermore, the analysis includes smoking status (yes/no). Finally, we included the geographic region of residency (Central Hungary, Southern Great Plain, Southern Transdanubia, Central Transdanubia, Western Transdanubia, Northern Great Plain, or Northern Hungary) based on the Nomenclature of territorial units for statistics (NUTS 2) to adjust our analysis for potential confounders resulting from territorial heterogeneity. All variables were self-reported.

### Statistical methods

In terms of categorical characteristics, Pearson’s chi-square test was performed to evaluate the differences between people who have been uptake breast screening within two years and who have been uptake it more than two years ago. To determine the variables that may have an impact on the uptake of screening, generalized linear model with logit link function regarding binomial probability distribution was executed. *P*-values and adjusted odds ratios were used to express the results. Stata Statistical Software (version 13.0, Stata Corp, College Station, TX, USA) was used for the statistical analysis, and *p* < 0.05 was considered significant.

## Results

The original sample size was 16,480, comprising 5,051 respondents from the 2009 dataset, 5826 participants from the 2014 dataset, and 5603 respondents from the 2019 dataset. In 2009, there were 971 women aged 45–65 (19%); the figures for 2014 and 2019 were 1048 (18%) and 1035 (18%), respectively. After combining the datasets (*n* = 3054), we removed respondents (*n* = 428; 14%) who did not respond to all relevant research-related questions, so the final sample size was 2,626.

### The pattern shown by descriptive statistics

As shown in Table 1, in terms of educational level, similar significant results (*p* < 0.001) were obtained in 2009, 2014 and 2019, with the highest prevalence of breast screening among those with tertiary education, followed by secondary education and finally primary education. Place of residence showed a significant association (*p* = 0.012) with breast screening attendance only in 2019, with those living in cities being more likely to attend screening. Regarding marital status, a significant relationship (*p* < 0.001) was observed in 2014 compared to 2009 and 2019. A higher number of participants who were married or had a partner attended screening than those who did not have a partner. In both 2009 (*p* = 0.001), 2014 (*p* < 0.001) and 2019 (*p* = 0.007), significant results were obtained when observing the perceived income of participants, with those who answered they have a good perceived income being more likely to be screened. In terms of self-perceived health status, significant results were found for screening attendance in 2014 (*p* = 0.009) and 2019 (*p* = 0.010), with those who said they had good self-perceived health status, attending breast screening more frequently. The factor of how much someone can do for their health showed a significant result in 2009 (*p* = 0.024) and 2014 (*p* = 0.001). People who said they could do a lot for their health were more likely to be screened. There was no significant difference in breast screening uptake between those with and without at least one chronic disease in any year. Examining the last meeting with the doctor, we found that both in 2009 (*p* < 0.001), 2014 (*p* < 0.001) and in 2019 (*p* = 0.001), those who have visited their doctor within a year were significantly more likely to participate in screening than those who had seen their doctor more than a year ago. Similar results were obtained, investigating the last meeting with a specialist; in all three years there were significant difference (*p* < 0.001) between the frequency of screening uptake among those who had been to a specialist within a year, and more than a year. In terms of smoking status, we found that both in 2009 (*p* < 0.001), 2014 (*p* < 0.001) and 2019 (*p* = 0.008) participants who were not smoking had higher frequency of screening attendance than smokers. There was territorial heterogeneity across regions in Hungary in all three years, however, statistically proven significant differences were not observed.

**Table 1:** Breast screening participation rate based on the EHIS study among the Hungarian women aged 45–65

Factors		2009 N=876			2014 *N*=950			2019 *N*=800			Merged sample *N*=2626		
		Attended breast screening in the past two years (N)	Attended breast screening in the past two years (%)	***p***-value	Attended breast screening in the past two years (N)	Attended breast screening in the past two years (%)	***p***-value	Attended breast screening in the past two years (N)	Attended breast screening in the past two years (%)	*p*-value	Attended breast screening in the past two years (N)	Attended breast screening in the past two years (%)	***p***-value
**Educational level**	Primary	129	54%	<0.001*	102	50%	<0.001*	75	47%	<0.001*	306	51%	<0.001*
	Secondary	373	72%		395	71%		307	67%		1,075	70%	
	Tertiary	92	77%		133	72%		133	72%		358	74%	
**Type of residence**	Urban	395	68%	0.640	449	68%	0.060	356	67%	0.012*	1,200	68%	0.005*
	Rural	199	67%		181	62%		159	58%		539	63%	
**Marital status**	Married or have a partner	366	68%	0.632	416	71%	<0.001*	354	66%	0.269	1,136	69%	0.001*
	Not have a partner	228	67%		214	58%		161	62%		603	62%	
**Perceived income**	Good	412	72%	0.001*	507	72%	<0.001*	443	66%	0.007*	1,362	70%	<0.001*
	Bad	182	60%		123	50%		72	54%		377	55%	
**Self-perceived health status**	Good	457	67%	0.477	555	68%	0.009*	458	66%	0.010*	1,470	67%	0.029*
	Bad	137	70%		75	56%		57	53%		269	62%	
**How much can you do for your health**	A lot	432	70%	0.024*	511	69%	0.001*	432	65%	0.166	1,375	68%	<0.001*
	Not so much	162	62%		119	56%		83	59%		364	60%	
**Chronic health problem**	Has at least 1	523	69%	0.056	389	66%	0.895	320	65%	0.552	1,232	67%	0.163
	Has none	71	60%		241	67%		195	63%		507	64%	
**Last meeting with a doctor**	<12 months	531	70%	<0.001*	562	69%	<0.001*	449	67%	0.001*	1,542	68%	<0.001*
	≥12 months	63	53%		68	50%		66	52%		197	51%	
**Last meeting with a specialist**	<12 months	454	73%	<0.001*	491	71%	<0.001*	403	69%	<0.001*	1348	71%	<0.001*
	≥12 months	140	55%		139	54%		112	52%		391	54%	
**Smoker**	Yes	155	59%	<0.001*	155	57%	<0.001*	130	57%	0.008*	440	58%	<0.001*
	No	439	72%		475	70%		385	67%		1,299	70%	
**Region (NUTS 2)**	Central Hungary	129	63%	0.055	141	65%	0.121	144	63%	0.698	414	63%	0.029*
	Southern Great Plain	92	74%		78	63%		79	72%		249	70%	
	Southern Transdanubia	49	58%		67	58%		54	63%		170	59%	
	Northern Great Plain	104	72%		123	74%		77	63%		304	70%	
	Northern Hungary	83	67%		81	69%		56	62%		220	67%	
	Central Transdanubia	66	66%		80	69%		54	68%		200	68%	
	Western Transdanubia	71	76%		60	64%		51	62%		182	67%	
**Year of the survey**	2009	-									594	68%	0.331
	2014										630	66%	
	2019										515	64%	

*Significant findings (*p* < 0.05)

### Breast screening participation rate within two years of survey respondents aged 45–65 based on the merged sample

In 2009 85% (*n* = 741), in 2014 90% (*n* = 851) and in 2019 87% (*n* = 699) of women said that they have attended breast screening at least once during their whole life. As shown in Table 1 in 2009 68% (*n* = 594), in 2014 66% (*n* = 630) and in 2019 64% (*n* = 515) of the respondents said that they have taken part in breast screening within the past two years (*p* = 0.331). The highest participation rate was found among those with a tertiary level of education (*n* = 358; 73%), which was followed by secondary (*n* = 1075; 70%) and primary (*n* = 306; 51%) education (*p* < 0.001). People living in the city had significantly higher attendance rate (*n* = 1200; 68%) than the ones who are from the village (*n* = 539; 63%) (*p* = 0.005). Those women who are married or have a partner had significantly higher screening attendance rate (*n* = 1136; 69%) than who do not have a partner (*n* = 603; 62%) (*p* = 0.001). People who said that they have good income had significantly higher participation rate (*n* = 1362; 70%) than people with worse income (*n* = 377; 55%) (*p* < 0.001). Attendance rate was higher in respondents with good self-perceived health status (*n* = 1470; 67%) than those with bad self-perceived health status (*n* = 269; 62%) (*p* = 0.029). Those who said that they could do much for their own health had higher participation rate (*n* = 1375; 68%) compared to those who stated that they could not do a lot for their health (*n* = 364; 60%) (*p* < 0.001). The frequency of screening attendance was higher – but not significantly – in the case of people with a chronic health problem (*n* = 1232; 67%), than the ones who did not have any (*n* = 507; 64%) (*p* = 0.163). Those respondents who visited their doctor within a year had significantly higher attendance rate (*n* = 1542; 68%) than the ones who did not visit their doctor in the previous year (*n* = 197; 51%) (*p* < 0.001). Residents who visited their specialist within twelve months had significantly higher participation rate (*n* = 1348; 71%), than the ones who visited their specialist more than a year ago (*n* = 391; 54%) (*p* < 0.001). Those respondents who are smoking had significantly lower attendance rate (*n* = 440; 58%) compared to those who are not (*n* = 1299; 70%) (*p* < 0.001). A significant territorial heterogeneity was observed, ranging between 59% and 70% (*p* = 0.029). The lowest participation rate was in Southern Transdanubia (*n* = 170; 59%) and the highest was in Southern Great Plain (*n* = 249; 70%) and Northern Great Plain (*n* = 304; 70%).

### The pattern shown by the generalized linear model with logit link function regarding binomial probability distribution

As shown in Table 2, in terms of educational level, we found significant association with screening attendance in all three years. Participants who had secondary or tertiary education had higher chance of participating on breast screening than residents with primary education. The type of residence did not show significant connection with screening participation in any of the years. Examining the three years, marital status had an impact on screening uptake in 2014. Those who were married or had a partner were more likely to attend on screening than the ones who did not have a partner. We found that perceived income had a significant effect in 2009 and 2014; residents with higher perceived income had greater odds of attend on screening. Self-perceived health status showed a significant association with screening uptake in 2009; those who said that their health status is good had higher chance of participate on screening. There were no significant association between the factor of how much one can do for her own health, the existence of at least one chronic problem and screening attendance in any of the years. Last meeting with a doctor had an impact on screening uptake in 2009 and 2014, while last meeting with a specialist had an effect in all three years; residents who had seen their doctor or specialist within a year had higher odds on participate on breast screening. Smoking status proved to be a significant influencing factor in 2014; non-smokers were more likely to be screened. Investigating Hungary’s regions, we found that in 2009 residents from Northern Great Plain and Western Transdanubia, in 2019 people from Southern Great Plain and Central Transdanubia had significantly higher odds of screening attendance than the ones who had been living in Central Hungary.

**Table 2:** Analysis of the factors which influence breast screening attendance among Hungarian women aged 45–65

Factors		2009 *N* = 876			2014 *N* = 950			2019 *N* = 800			Merged sample		
		Adjusted Odds Ratio	95% Confidence Intervals		Adjusted Odds Ratio	95% Confidence Intervals		Adjusted Odds Ratio	95% Confidence Intervals		Adjusted Odds Ratio	95% Confidence Intervals	
**Educational level**	Secondary/Primary	2.28*	1.60	3.25	1.90*	1.31	2.76	1.94*	1.29	2.91	1.97*	1.60	2.44
	Tertiary/Primary	2.82*	1.61	4.91	1.75*	1.08	2.86	2.45*	1.43	4.18	2.24*	1.67	3.00
**Type of residence**	Urban/Rural	0.88	0.63	1.23	1.03	0.74	1.43	1.31	0.93	1.83	1.08	0.89	1.31
**Marital status**	Married or have a partner/Not have a partner	0.93	0.68	1.28	1.46*	1.09	1.97	1.07	0.77	1.50	1.16	0.97	1.38
**Perceived income**	Good/Bad	1.57*	1.16	2.20	1.82*	1.28	2.59	1.27	0.82	1.97	1.54*	1.25	1.90
**Self-perceived health status**	Good/Bad	0.63*	0.41	0.97	1.03	0.65	1.64	1.50	0.91	2.49	0.97	0.74	1.26
**How much can you do for your health**	A lot/Not so much	1.43	0.99	2.06	1.12	0.77	1.64	0.92	0.59	1.44	1.15	0.92	1.43
**Chronic health problem**	Has at least 1/Has none	1.10	0.68	1.77	0.84	0.60	1.17	1.04	0.73	1.48	0.95	0.77	1.18
**Last meeting with the doctor**	< 12 months/≥12 months	1.65*	1.05	2.61	2.08*	1.37	3.16	1.53	1.00	2.36	1.77*	1.39	2.27
**Last meeting with the specialist**	< 12 months/≥12 months	1.93*	1.37	2.71	1.88*	1.35	2.63	1.93*	1.34	2.79	1.88*	1.54	2.28
**Smoker**	Yes/No	0.75	0.54	1.04	0.68*	0.50	0.94	0.79	0.56	1.11	0.74*	0.61	0.89
**Region (NUTS 2)**	Central Hungary	Reference											
	Southern Great Plain	1.59	0.94	2.68	0.90	0.55	1.48	2.12*	1.25	3.62	1.45*	1.08	1.95
	Southern Transdanubia	0.86	0.50	1.51	0.81	0.49	1.35	1.31	0.76	2.26	0.99	0.73	1.34
	Northern Great Plain	1.87*	1.14	3.08	1.61	1.00	2.60	1.15	0.71	1.85	1.53*	1.16	2.02
	Northern Hungary	1.23	0.74	2.06	1.46	0.86	2.49	1.40	0.80	2.44	1.40*	1.03	1.89
	Central Transdanubia	1.08	0.63	1.83	1.25	0.74	2.10	1.83*	1.02	3.28	1.36	1.00	1.86
	Western Transdanubia	2.05*	1.14	3.68	0.99	0.57	1.71	1.27	0.72	2.23	1.35	0.98	1.86
**Year of the survey**	2014/2009	-									0.83	0.67	1.02
	2019/2009										0.72*	0.57	0.89

Reference groups are underlined; *Significant findings (*p* < 0.05)

### Results of the generalized linear model with logit link function regarding binomial probability distribution based on the merged sample

From 2009 to 2014 (AOR = 0.83 [0.67–1.02]) the attendance of breast screening not decreased significantly, while from 2014 to 2019 (AOR = 0.72 [0.57–0.89]) it reduced significantly (Table 2). Both secondary (AOR = 1.97 [1.60–2.44]) and tertiary educational level (AOR = 2.23 [1.67-3.00]) was a crucial protective factor since those with it were more likely to participate in the screening than people with primary education. No significant association was found between the type of residence and the breast screening attendance (AOR = 1.08 [0.89–1.31]). We found that marital status does not have an effect on screening participation (AOR = 1.16 [0.97–1.38]). Residents with higher perceived income were more likely to attend screening than people with lower income (AOR = 1.54 [1.25–1.90]). There was no significant association between self-perceived health status (AOR = 0.97 [0.74–1.26]), the factor of how much one can do for her own health (AOR = 1.15 [0.92–1.43]) and screening participation. Both the last meeting with the doctor and with the specialist showed a significant correlation with the attendance on screening. Respondents who visited their doctor in a year had more than 1.5 times (AOR = 1.77 [1.39–2.27]), and people who visited their specialist had almost 2 times greater odds (AOR = 1.88 [1.54–2.28]) of attending a breast screening. Those women who are smoking had a lower chance of screening participation (AOR = 0.74 [0.61–0.89]) compared to non-smokers. Examining the Hungarian regions, we found significant associations as well. Residents from Southern Great Plain (AOR = 1.45 [1.08–1.95]), Northern Great Plain (AOR = 1.53 [1.16–2.02]) and Northern Hungary (AOR = 1.40 [1.03–1.89]) had significantly higher odds of screening attendance than those from Central Hungary.

## Discussion

The majority of European nations have implemented mammography-based screening programs as a result of research demonstrating mammography screening’s ability to lower breast cancer mortality. Despite recommendations and programs, screening age groups also vary from country to country [19]. However, participation of breast cancer screening varies widely across Europe, ranging from just under 30% to over 90% [19].

According to Eurostat data, breast screening participation among women aged 50–69, in 2019 was 68% in Slovenia, 77% in the Czech Republic, 63% in Croatia and 54% in Slovakia [20]. Based on our study, the proportion of people attending breast screening was considered as low in 2009, at 68%, and this low proportion has further decreased to 64% in 2019 in Hungary.

In our study the European Health Interview Survey was used to conduct an analysis of breast screening uptake and its determining factors among Hungarian women aged 45–65. The findings suggest that the lifetime prevalence was high; however, the recommended two years’ participation rate could be considered as lower compared to the expected 70–75%, defined by The European Guidelines for Quality Assurance in Breast Cancer Screening and Diagnosis [21].

In addition, we were able to identify the most imperative factors which were associated with breast screening attendance. Based on our study the variable with the highest odds ratio, therefore, with the most impact was educational level, followed by the last meeting with a specialist, the last meeting with a doctor, perceived income, region, and finally smoking status. Analyzing the possible determining factors, we observed that higher educational level, higher income, and more frequent meeting with the doctor and with the specialist appeared as protective factors of breast screening attendance. Women with secondary and tertiary educational level, perceived good financial status and more frequent appointments with their doctor had greater chance to participate in breast screening within two years. Territorial heterogeneity was noticed, the highest attendance rate were in Southern Great Plain and in Northern Great Plain, while the lowest was in Southern Transdanubia. Accessibility of the screening is probably playing a key role in these differences. Based on our results, the risk groups that should be given high priority are people with lower educational level, worse financial status and less frequent appointments with the doctor and specialist. Education level contributing to breast cancer screening was in line with the literature [22–24]. In the case of financial status we found that perceived financial status is not always related to the attendance of screening participation [25]. There are other factors, which have an influence on the attendance on screening, for example women’s attitude or previous knowledge about breast cancer screening [26], although in our study we are not able to examine these factors.

Several strategies could increase screening participation, such as health promotion screening buses. Those are used to make organized public health screening available to people living in communities far from screening centers, also to reduce health inequalities [27]. Currently mammography is not yet available on buses in Hungary [27]; however, breast screening with buses is an accepted method for mammography screening, since it’s a common method in other countries, such as in Ontario, Canada [26]. As part of the Ontario Breast Screening Program, the Mobile Cancer Screening Program offers mammography every two years for women aged 50 to 74 [28]. In the Netherlands, the Dutch National Breast Cancer Screening Program is intended for females 50 to 75 years old. Women in this age group are invited for a mammogram every two years [29]. Furthermore, different kind of interventions, included promotional materials – such as posters –, group and one-on-one interventions, written resources and workshops could also help to increase breast screening participation [30].

### Strengths and limitations

Although the primary objective of the European Health Interview Survey was not to determine the prevalence of attendance on breast cancer screening, the most important factors of screening participation were identified in our analysis. Since the same method was used in all three surveys, the data could be compared to one another and used in an aggregated form. Also, the number of participants overall and each category made the statistical analysis feasible. However, it is important to take into consideration that using self-reported questionnaires may have resulted in under-representation in the results. The database only included data on respondents due to the methodological nature of the data collection, while no information was gathered about those who rejected participation. It should also be taken into consideration that we cannot know which specialist the respondents have seen. In addition, our study focused on people aged between 45 and 65, which is the recommended target group for screening in Hungary, however, data described by Eurostat is refer to people aged 50–69. Thus, caution is advised when comparing the Hungarian participation rate with the ones in neighboring countries. Finally, it is also important to note that we did not have information on whether or not the respondent had received breast cancer screening as part of the organized screening.

## Conclusions

The proportion of women aged 45–65 in Hungary who has participated in breast screening is below the acceptable level, and the trend showed a decreasing change between 2009 and 2019. Therefore, health policy makers should further increase their efforts to increase the proportion of those who attend the screening, especially among the identified risk groups, as well as tackling regional inequalities.

## Data Availability

The data that support the findings of this study are available from the Hungarian Central Statistical Office but restrictions apply to the availability of these data, which were used under license for the current study, and so are not publicly available. The data are, however, available from the corresponding author upon reasonable request and with the permission of from the Hungarian Central Statistical Office.

## References

[1] Fahrin RA, Calum D, Khaoula B, Abdourahmane N, Hannah K (2022). Disability and participation in breast and cervical Cancer screening: a systematic review and Meta-analysis. Int J Environ Res Public Health.

[2] Sung H, Ferlay J, Siegel RL, Laversanne M, Soerjomataram I, Jemal A (2021). BrayGlobal Cancer Statistics 2020: GLOBOCAN estimates of incidence and Mortality Worldwide for 36 cancers in 185 countries. CA Cancer J Clin.

[3] WHO position paper on mammography screening. World Health Organization (WHO). 2014. Available online: https://www.who.int/publications/i/item/who-position-paper-on-mammography-screening. Accessed 12 Mar 2023. 25642524

[4] Cancer.Net: Breast Cancer: Statistics. 2023. Available online: https://www.cancer.net/cancer-types/breast-cancer/statistics. Accessed 18 Mar 2023.

[5] Jansen EEL, Zielonke N, Gini A, Anttila A, Segnan N, Vokó Z, Ivanuš U, McKee M, de Koning HJ, de Kok IMCM (2020). Effect of organised cervical cancer screening on cervical cancer mortality in Europe: a systematic review. Eur J Cancer.

[6] Shah R, Rosso K, Nathanson SD (2014). Pathogenesis, prevention, diagnosis and treatment of breast cancer. World J Clin Oncol.

[7] Hosseinzadeh M, Eivazi Ziaei J, Mahdavi N, Aghajari P, Vahidi M, Fateh A (2014). Risk factors for breast cancer in iranian women: a hospital-based case-control study in Tabriz, Iran. J Breast Cancer.

[8] Breast cancer. World Health Organization (WHO)., 2021. Available online: https://www.who.int/news-room/fact-sheets/detail/breast-cancer. Accessed 6 Mar 2023.

[9] Breast cancer statistics. World Cancer Research Fund. 2022. Available online: https://www.wcrf.org/cancer-trends/breast-cancer-statistics/. Accessed 26 Feb 2023.

[10] Béres E, Nagy J, Tóth J, Árkosy P, Sipka S (2022). Standardized data on the incidence and mortality of female and male breast cancers in Hungary between 2000 and 2016. Orv Hetil.

[11] What Is Breast Cancer Screening? Centers for Disease Control and Prevention (CDC). 2022. Available online: https://www.cdc.gov/cancer/breast/basic_info/screening.htm. Accessed 20 Feb 2023.

[12] American Cancer Society: American Cancer Society Recommendations for the Early Detection of Breast Cancer. 2022. Available online: https://www.cancer.org/cancer/breast-cancer/screening-tests-and-early-detection/american-cancer-society-recommendations-for-the-early-detection-of-breast-cancer.html. Accessed 28 Feb 2023.

[13] Mishra GA, Pimple SA, Mittra I, Badwe RA (2021). Screening for breast cancer. Cost-effective solutions for low- & middle-income countries. Indian J Med Res.

[14] Screening ages and frequencies. European Commission. 2023. Available online: https://healthcare-quality.jrc.ec.europa.eu/ecibc/european-breast-cancer-guidelines/screening-ages-and-frequencies. Accessed 2 Mar 2023.

[15] Council of the European Union: Council Recommendation on strengthening prevention through early detection: A new EU approach on cancer screening replacing Council Recommendation 2003/878/EC, 2022. Available online: https://data.consilium.europa.eu/doc/document/ST-14770-2022-INIT/en/pdf. Accessed 6 Apr 2023.

[16] Information about breast screening. National Public Health Centre. 2020. Available online: https://www.nnk.gov.hu/index.php/component/content/article/179-projektek/efop-1-81/informaciok-az-efop-1-8-1-projekten-belul/618-informaciok-az-emloszuresrol. Accessed 6 Apr 2023.

[17] Breast screening. National Public Health Centre. 2020. Available online: https://www.nnk.gov.hu/index.php/szuresiranyitasi-foosztaly/nepegeszsegugyi-celu-szuresi-koordinacios-osztaly/emloszures. Accessed 6 Apr 2023.

[18] Hungarian Central Statistical Office. European Health Interview Survey. Available online: https://www.ksh.hu/elef. Accessed 6 Feb 2023.

[19] Rafael C, Michael H, Hermann B (2023). Breast cancer screening programmes and self-reported mammography use in european countries. Int J Cancer.

[20] 66% of women in the EU aged 50–69 got a mammogram. Official website of the European Union. 2021. https://ec.europa.eu/eurostat/web/products-eurostat-news/-/edn-20211025-1. Accessed 23 July 2023.

[21] Perry N, Broeders M, de Wolf C, Törnberg S, Holland R, von Karsa L. European Guidelines for Quality Assurance in Breast Cancer Screening and Diagnosis. 4th ed. Luxembourg: Office for Official Publications of the European Communities. 2006. 10.1093/annonc/mdm481 18024988

[22] Hale K, Owen OD, Tom VO (2018). What Explains Education Disparities in Screening Mammography in the United States? A Comparison with The Netherlands. Int J Environ Res Public Health.

[23] Helen IM, Nancy B, Michele LT, Sally WV, Barry IG (2007). Which women aren’t getting mammograms and why? (United States). Cancer Causes Control.

[24] Tam TD, Al HAK, Mohamed GAK, Salha BAB, Nabila AM, Mariam A, Rajvir S, Sofia C, Tak F. Do socioeconomic factors influence breast cancer screening practices among Arab women in Qatar? BMJ Open. 2015;5(1):e005596. 10.1136/bmjopen-2014-005596 PMC4305075 25613951

[25] Maria MG, Jacopo L, Marco B, Maria PF, Roberta S, Walter R, Gianfranco D (2018). Organized screening programmes for breast and cervical cancer in 17 EU countries: trajectories of attendance rates. BMC Public Health.

[26] Juan JMS, María JP, Elisa MGA, Juan RM (2021). Non-participation in breast Cancer screening in Spain and potential application in the Present and Future: A Cross Sectional Study. Cancers (Basel).

[27] “Helybe visszük a szűrővizsgálatokat”. National Public Health Centre. Available online: https://www.nnk.gov.hu/index.php/szuresiranyitasi-foosztaly/szuroprogram-iranyitasi-osztaly/helybe-visszuk-a-szurovizsgalatokat-program (accessed on 16 March 2023).

[28] Mobile Cancer Screening Coach. Hamilton Health Sciences. Available online: https://www.hamiltonhealthsciences.ca/areas-of-care/cancer-care/mobile-cancer-screening-coach/. Accessed 2 Apr 2023.

[29] Breast Cancer Screening Programme: National Institute for Public Health and the Environment. 2023. Available online: https://www.rivm.nl/en/breast-cancer-screening-programme. Accessed 16 Mar 2023.

[30] Natasha A, Joanne L (2019). The impact of breast cancer awareness interventions on breast screening uptake among women in the United Kingdom: a systematic review. J Health Psychol.

